# Robotic Right Lower Lobectomy in a Patient With V2 and V4+5 Pulmonary Vein Variation Merging Into the Lower Pulmonary Vein: A Case Report

**DOI:** 10.7759/cureus.57491

**Published:** 2024-04-02

**Authors:** Rurika Hamanaka, Makoto Oda

**Affiliations:** 1 Shin-Yurigaoka General Hospital, Department of Thoracic Surgery, Kawasaki, JPN

**Keywords:** 3d-ct angiography, right lower, incomplete fissures, robotic surgery, pulmonary vein variation

## Abstract

Several variations of pulmonary vein (PV) branching patterns exist. Since robot-assisted thoracoscopic surgery (RATS) is performed with magnified vision, it is crucial to carefully identify the running pattern of blood vessels before and during surgery. We present a case of a 77-year-old male patient with right lower lobe lung cancer. Right lower lobectomy via RATS was scheduled. Chest CT before surgery confirmed that the middle lobe PV (V^4+5^) merged with the inferior PV. Three-dimensional multidetector CT (3D-MDCT) subsequently confirmed that not only V^4+5^ but also the posterior segmental vein of the upper lobe (V^2^) merged with the inferior PV. We should have taped the lower lobe PV only, but we also taped the V^2 ^and the middle lobe vein. However, since the oblique fissure was separated before cutting the taped blood vessel, the cutting of the blood vessel to be preserved was avoided.

Surgeons should have a detailed understanding of the running patterns of pulmonary blood vessels before surgery to perform the procedure safely. Preoperative 3D-MDCT is useful for identifying the running pattern of blood vessels. An abnormality involving V^2^ and V^4+5^ merging into the inferior PV can also occur; hence, during right lower lobe resection, by dividing the lower lobe PV after the oblique fissure division, the surgeon can avoid unexpected transection of anomalous PVs that should be preserved.

## Introduction

Various running patterns of the pulmonary vein (PV) have been described [1,2,3]. In most patients, the right PV is the superior PV, which joins the upper lobe PV and the middle lobe PV, and the inferior PV, which joins the lower lobe PV. However, it has been reported that 14% of patients demonstrate other patterns. In one study, 10% of patients had three branches on the right side, and 4% of patients had four or five branches [2]. In other reports, V^4^ and V^5^ merged into the inferior PV in 4.8% of cases, while one merged with the superior PV and the other merged with the inferior PV in 0.8% of cases [1]. If the running pattern of the PV is not carefully confirmed before and during surgery, the surgeon risks cutting the PV that should be preserved, and this may result in pulmonary congestion [4].

Robot-assisted thoracoscopic surgery (RATS) has been gaining rapid popularity. RATS is a minimally invasive procedure, and it is predicted that the number of RATS surgeries for cases with PV abnormalities will significantly increase in the future. RATS allows surgeons to perform surgery with a magnified field of view and high-definition 3D field of view, thereby making the procedures safer [5]. However, on the other hand, the enlarged field of view widens the area outside the field of view, making it difficult to judge the surrounding scenario. Although this is more likely to occur with video-assisted thoracoscopic surgery (VATS) than with open surgery, this drawback is more pronounced in RATS since the surgery is performed with an enlarged field of view than in VATS. Therefore, it is vital to identify the running pattern of blood vessels before and during surgery; three-dimensional multidetector computed tomography (3D-MDCT) is extremely useful for this purpose. Preoperative 3D-MDCT can help identify the running pattern of blood vessels and thus help avoid dangerous misidentification during the operation.

We discuss a case of a patient with lung cancer who underwent a right lower lobectomy via RATS in which V^2^ and the middle lobe vein joined the inferior PV.

## Case presentation

**Figure 1:**
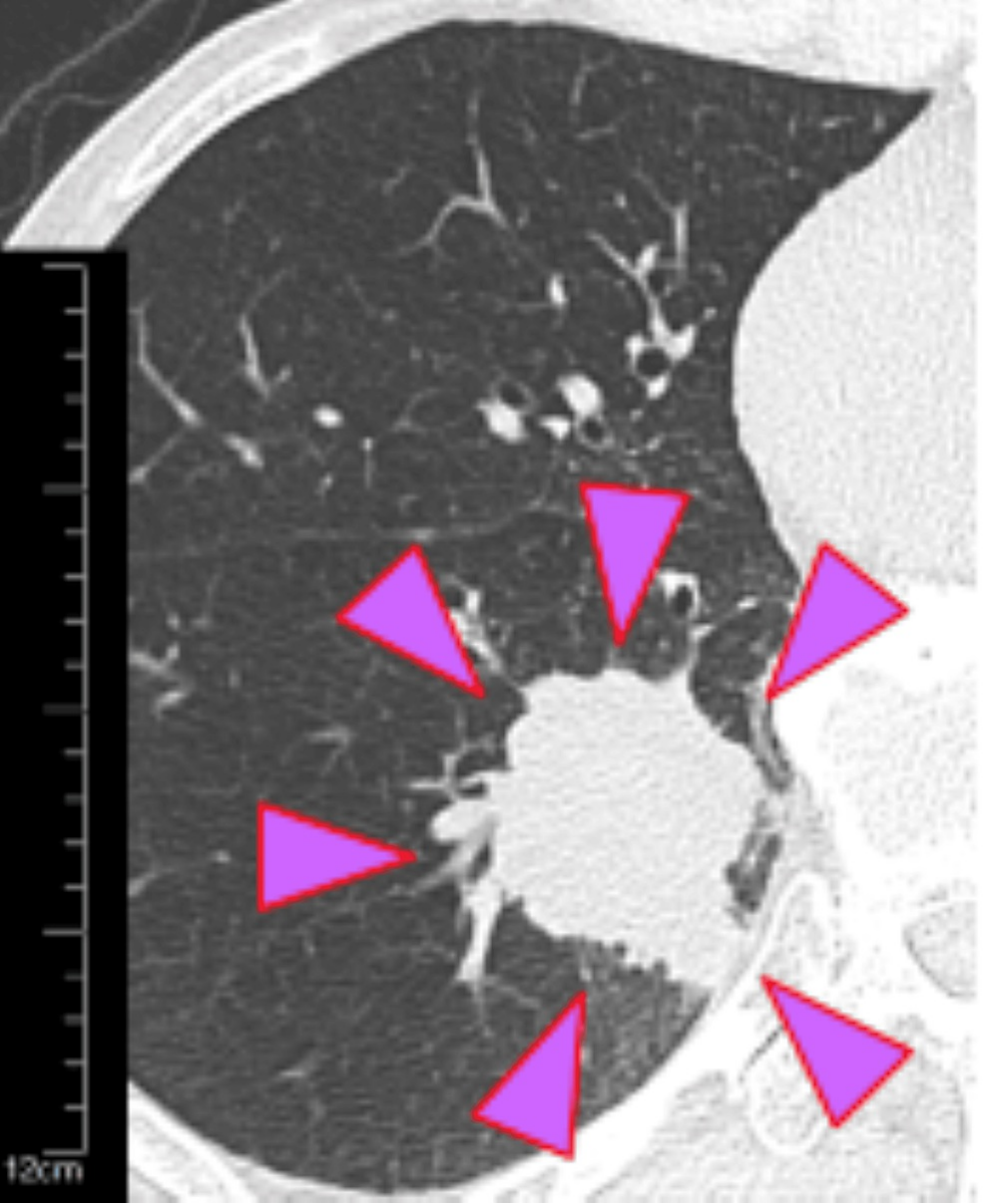
Chest CT of the patient showing a right lower lobe lung mass A tumor with a total lesion diameter of 3.8 cm and a solid component diameter of 3.8 cm was found in S9-10 of the right lower lobe CT: computed tomography

A 77-year-old male was found to have a right lower lobe lung mass on chest CT during a clinical examination (Figure 1).

**Figure 2:**
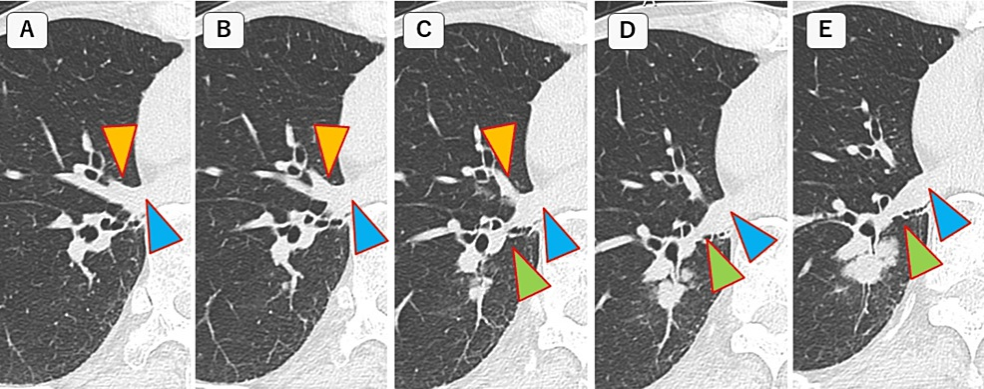
CT findings before surgery (A-E) Serial CT image with a 1 mm thick slap from cephalad to caudal. The yellow arrow indicates the middle lobe pulmonary vein (PV), the green arrow shows the lower lobe PV, and the blue arrow shows the inferior PV. The middle lobe PV joins the inferior PV. However, these images did not show the posterior segmental vein of the upper lobe (V^2^) merging into the inferior PV CT: computed tomography

The tumor was diagnosed as lung cancer by bronchoscopic biopsy, and a right lower lobectomy by RATS was scheduled. Chest CT before surgery confirmed that the middle lobe PV merged with the inferior PV (Figure 2).

**Figure 3:**
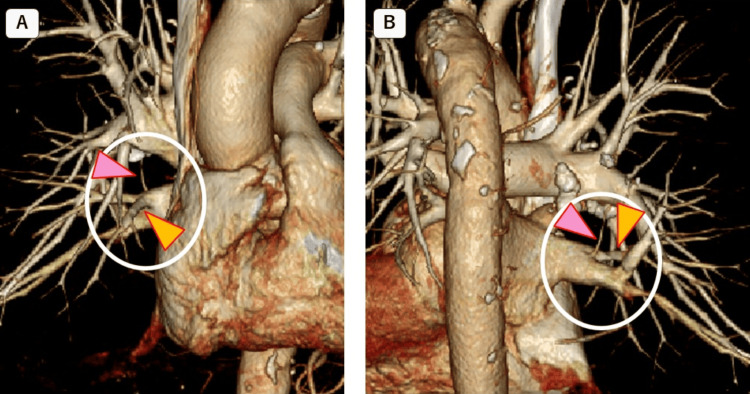
3D-MDCT before surgery (A) 3D-MDCT frontal view. (B) 3D-MDCT back view. The inferior pulmonary vein (PV) is shown in the white circle. The pink arrow shows the posterior segmental vein of the upper lobe (V^2^) and the yellow arrow shows the middle lobe PV; both PVs joining the inferior PV can be clearly seen 3D-MDCT: three-dimensional multidetector computed tomography

A 3D-MDCT subsequently confirmed that not only the middle lobe PV but also V ^2^ merged with the inferior PV (Figure 3).

The patient underwent surgery via total portal RATS using the da Vinci Xi surgical system [6]. The oblique fissure was observed to be an incomplete fissure. The pulmonary ligament and mediastinal lymph nodes were dissected. The A ^6^ pulmonary artery and station 11 lymph nodes were confirmed posterior to the hilum, and the incompletely lobated oblique fissure between the upper and lower lobes was divided with da Vinci staplers. The A ^6-10^ pulmonary artery and the lower lobe bronchus were respectively divided. The inferior PV was examined from the diaphragm surface side, and a vessel loop was circumferentially placed around the lower lobe PV. The incompletely lobated oblique fissure between the middle and lower lobes was divided first, and the running pattern of the PV was then confirmed from the fissure side. The running pattern of the PV was then reconfirmed, and it was found that V ^2^ and the middle lobe PV, which had joined the inferior PV, were taped together. Only the lower lobe PV was retaped and divided, and lower lobectomy was thus accomplished. The operation lasted for two hours and 31 minutes. The amount of bleeding was minimal, and the postoperative course was uneventful. The chest tube was removed on the first day postoperatively, and the patient was discharged on the following day. The pathological diagnosis was pT2aN2M0, stage IIIA squamous cell carcinoma, and both the total lesion diameter and solid component diameter were 4.0 cm.

## Discussion

Although the middle lobe PV usually merges with the superior PV, these PVs merge with the inferior PV in some cases [1]. In one study, V^4^ and V^5^ merged into the inferior PV in 4.8% of cases, and one merged with the superior PV and the other merged with the inferior PV in 0.8% of cases [1]. In this case, not only the middle lobe PV but also the V^2^ joined the inferior PV. To the best of our knowledge, this is the first report describing a right lower lobectomy via RATS in such a case.

To ensure surgical safety, the surgeon must have a clear understanding of the running pattern of each patient's blood vessels before surgery. In one report [4], the operation was performed without recognizing the anomalous PV junction, and the lobe to be preserved had to be excised. In another report [7], the operation was performed without recognizing the anomalous PV junction, and the PV had to be reconnected. Thus, it is very important to understand the blood vessel running pattern before the operation. PV abnormalities are difficult to detect using transverse section CT; however, 3D-MDCT makes it easier to detect abnormalities [8]. We perform 3D-MDCT before surgery in all patients scheduled for lobectomy when contrast-enhanced CT is feasible and confirm the running pattern of the pulmonary arteries and veins. It has been reported that 97.7% of pulmonary arteries were accurately identified when 3D-MDCT was performed [9]. However, data are scarce in the literature about the accuracy of 3D-MDCT in correctly detecting PVs. It is often difficult to grasp the detailed running pattern of the PV with normal CT alone, and 3D-MDCT is extremely helpful in ensuring the safety of the surgery and reducing surgical time. Although we have found no reports about the utility of 3D-MDCT in RATS, some reports have described the effectiveness of 3D-MDCT in VATS [2,7,10-12].

Akiba et al. and Irene et al. have stated that it is more important to understand the running pattern of blood vessels by 3D-MDCT in VATS than in open surgery because VATS is performed by two-dimensional vision and reduced tactile sensation, unlike open chest surgery [2,10]. According to Hagiwara et al., gaining a detailed understanding of surgical anatomy and simulation by using imaging modalities before surgery contributes to the safety of VATS. In addition, research has shown that when 3D-MDCT is performed in VATS, the surgical time is significantly shortened, the amount of bleeding is reduced, and postoperative complications are reduced [11].

Although RATS is performed with 3D vision, its field of view is magnified, and it is therefore more important to understand the running of blood vessels before and during surgery in RATS than in VATS. Because of this magnified field of view, it is more difficult to determine the surrounding situation in RATS than in VATS or open surgery, and there is a risk of cutting blood vessels that should be preserved. Particular attention should be paid to cases with incomplete lobation [13]. In our case, although the anomalous running pattern of the middle lobe PV was identified before the operation, the middle lobe PV and V^2^ that should be preserved were encircled by vessel tape together with the lower lobe PV, and there was a risk of dividing the middle lobe PV. Fortunately, we transected only the lower lobe PV by reconfirming the running pattern of the PVs after the oblique fissure was divided.

## Conclusions

We described a case involving unusual PV drainages where the middle lobe PV and V2 merged into the inferior PV, which underwent a right lower lobectomy via RATS without any complications. By dividing the lower lobe PV after the oblique fissure division, especially in the right lower lobectomy, surgeons can avoid unexpected transection of anomalous PVs that should be preserved in robotic lobectomy with a magnified field of view.
